# The impact of sterile inflammation in acute liver injury

**DOI:** 10.18053/jctres.03.2017S1.003

**Published:** 2017-02-12

**Authors:** Benjamin L. Woolbright, Hartmut Jaeschke

**Affiliations:** Department of Pharmacology, Toxicology & Therapeutics, University of Kansas Medical Center, Kansas City, Kansas, United States

**Keywords:** sterile inflammation, liver, neutrophil, monocyte, mechanisms, cholestasis, ischemia, reperfusion, acetaminophen

## Abstract

**Background**: The liver has a number of functions in innate immunity. These functions predispose the liver to innate immune-mediated liver injury when inflammation goes unchecked. Significant progress has been made in the last 25 years on sterile inflammatory liver injury in a number of models; however, a great deal of controversy and many questions about the nature of sterile inflammation still exist.

**Aim**: The goal of this article is to review sterile inflammatory liver injury using both a basic approach to what constitutes the inflammatory injury, and through examination of current models of liver injury and inflammation. This information will be tied to human patient conditions when appropriate.

**Relevance for patients**: Inflammation is one of the most critical factors for managing in-patient liver disease in a number of scenarios. More information is needed for both scientists and clinicians to develop rational treatments.

## 1. Introduction

Liver associated morbidity and mortality is a major concern globally. The liver has a number of important functions in the body. One of the major functions of the liver is related to innate immunity, as the liver holds the largest macrophage population in the body, termed Kupffer cells (KCs). Diseases that result in acute liver injury, activate the liver’s innate immune functions, sometimes pathologically, even in the absence of an infection. Activation of KCs in the liver results in release of reactive oxygen species (ROS), and secretion of an array of cytokines that recruit other potentially cytotoxic inflammatory cells, including neutrophils, monocytes, T-cells, and NK/NKT cells [1]. The idea of innate immune-mediated liver injury in the absence of an infection has become a highly studied topic in liver biology over the last 25 years. While in some models there is nearly universal agreement that inflammation is a direct, pathological component of the injury process, there are other models wherein there is considerable debate over the role of sterile inflammation [2-5]. In a number of diseases, it is becoming increasingly understood that inflammation may actually play a beneficial, or at least a necessary role, as both acute and chronic liver failure can dramatically increase susceptibility to infection. The purpose of this review is to discuss mechanisms of sterile inflammatory liver injury and further discuss the role of sterile inflammation in clinically relevant models of acute liver injury.

## 2. Sterile inflammation – mechanisms of inflammation and injury

### 2.1 Release of damage associated molecular patterns

Sterile inflammation is a common outcome of a number of different clinical liver disorders. Sterile inflammation occurs in solid organs, such as the liver, when an organized inflammatory response occurs in the absence of any infection. While infectious inflammatory responses are driven by the detection and engulfment of infectious species, or pathogen associated molecular patterns (PAMPs), such as lipopolysaccharide and flagellin, sterile inflammatory responses are driven by local release of damage associated molecular patterns (DAMPs) from dying, necrotic cells [1, 6]. As cells die, their plasma membrane becomes permeable to intracellular contents [7]. In the case of outright necrosis, rupture of the cell occurs, and intracellular contents, or DAMPs, spill into the surrounding area. In the classic model of sterile inflammation these molecules then bind to pattern recognition receptors such as Toll-like receptors (TLRs), which induce pro-inflammatory cytokine formation [3,6,8]. Other DAMPs can either prime, or activate the multimeric protein complex called the inflammasome [9,10]. A number of damage associated molecular patterns have been identified including: ATP [11], high mobility group box-1 (HMGB1) [12], mitochondrial DNA and nuclear DNA [13,14], uric acid [15], hyaluronan [16], histones [17, 18] and more. Additionally, some molecules, such as bile acids (BA), have a number of similar qualities to known DAMPs, and are released during cell death [19]. Moreover, as necrosis results in total cellular breakdown, any number of cellular components could also be functioning as DAMPs. Thus, the sterile inflammatory response during liver injury is initiated by breakdown of the hepatocyte membrane and release of these constituents. The presence and the detection of these molecules drives the inflammatory response.

### 2.2 Activation of Kupffer cells

KCs are an endogenous macrophage population present in the sinusoidal space of the liver vasculature. Release of DAMPs activates a number of potential receptors on KCs. KCs express TLRs including TLR3, TLR4, TLR9 and more [20]. Additionally, KCs express purinergic receptors [21], the hyaluronan receptor CD44 [22], the receptor for advanced glycation end products (RAGE), which acts as a receptor for HMGB1 [23], and potentially other unknown receptors that also mediate these effects. Binding of these receptors serves to activate KCs resulting in local production of a number of cytokines and chemokines, in addition to the NF-κB pathway which causes upregulation of a multiple pro-inflammatory proteins [20]. Release of TNF-α from KCs can also activate NF-κB in hepatocytes, which upregulates adhesion molecules such as intercellular adhesion molecule-1 (ICAM-1) in models of liver injury [24]. In addition, in mice, many of these cytokines and chemokines such as macrophage inflammatory protein-2 (MIP-2), mouse keratinocyte factor (mKC), and macrophage chemoattractant protein-1 (MCP-1) have their own receptors i.e. CXC chemokine receptor 1 (CXCR1), CXC chemokine receptor 2 (CXCR2), and C-C chemokine receptor 2 (CCR2), respectively, all of which have been implicated in specific liver diseases [25-30]. Parallel activation of a number of these pathways likely occurs and amplifies the signals. All of these agents serve to increase recruitment of other inflammatory cells such as neutrophils and monocytes in addition to stimulating other resident macrophages.

Activation of TLR signaling on KCs also initiates the two-step process responsible for activating the inflammasome [31]. The inflammasome is a multimeric complex of proteins that initiates the activation of caspase-1 from pro-caspase 1, and subsequent cleavage of pro-interleukin-1ß (pro-IL-1ß) to IL-1ß, which serves as a potent pro-inflammatory cytokine [9]. While a number of different proteins can initiate formation of an inflammasome-like complex, the classical and most studied example of the inflammasome in the liver is the Nalp3 inflammasome [3]. Activation of TLR4, as an example, increases expression of pro-IL-1ß through the NF-κB pathway [8]. When KCs encounter DAMP signals, such as ATP, which activates the purinergic receptor 2X7 (P2X7), a ligand-gated ion channel, it causes release of intracellular potassium and activation of caspase-1 by the NLRP3 inflammasome. The activated caspase-1 cleaves pro-IL-1ß to form IL-1ß and then IL-1ß is released into the blood [9]. The NLRP3 inflammasome has been implicated in a number of models of liver injury, and will be discussed further in the context of specific diseases.

### 2.3 Initiation of inflammatory cell death – cytotoxic mediators of inflammation

Increased blood levels of cytokines and chemokines released from KCs result in rapid recruitment of multiple innate immune cells to the liver. The discussion will be mainly focused on the two cell populations that accumulate most commonly and in the highest numbers: neutrophils and monocytes/monocyte-derived macrophages. A brief section will highlight potential roles of other inflammatory cells as well. The liver recruits high numbers of both cell populations rapidly in response to injury. In models such as cholestatic liver injury [32,33], acetaminophen- (APAP) induced liver injury [34], hepatic ischemia-reperfusion liver injury [35], binge alcohol administration to chronically alcohol fed mice [36], and endotoxemia [37] there is rapid recruitment of neutrophils to the liver within 3-9 hours post initiation of toxicity. Monocytes typically follow shortly afterwards in some of these models [28]; however, it should be noted that KCs can also initiate toxicity and are endogenously present. Specific toxicity mechanisms of these two populations will be discussed in detail.

#### 2.3.1 Kupffer cells and monocyte-derived macrophages

KCs are endogenously present in the liver and are a primary innate immune cell type responsible for considerable bacterial, fungal, and viral clearance [38,39]. However, over-activation of KCs can be detrimental to hepatocyte function. KCs produce ROS through the enzyme NADPH oxidase (NOX2). Upon stimulation, KCs induce a vascular oxidant stress as evidenced by major increases in extracellular glutathione disulfide [40]. This is directly linked to hepatocyte cell death through activation of complement [41]. In support of these data, inactivation or removal of KCs with a number of differrent methods including administration of methyl palmitate, gadolinium chloride, or clodronate liposomes are protective against injury [40,42-46]. Given its established role in numerous diseases, release of ROS remains the most likely mechanism of toxicity from KCs.

In addition to production of ROS, KCs are frequently noted as potent producer of tumor necrosis factor-α (TNF-α), despite the fact that in a majority of liver diseases the actual increase in serum TNF-α levels is very limited. While there are models that are unequivocally mediated by release of TNF-α from KCs [47,48], in a majority of models, the release of TNF-α alone is insufficient to induce hepatocyte apoptosis. This is due to the fact that hepatocytes are highly resistant to TNF-α, and only undergo TNF-α mediated cell death in the presence of inhibition of nuclear factor kappa light chain enhancer of B cells (NF-κB) [49]. Human hepatocytes and human hepatocyte-like cell lines may be even more resistant to TNF-α, as they require even higher concentrations of the cytokine to induce apoptosis [50,51]. As such, TNF-α is likely less than oxidative stress to be relevant as a cytotoxic mediator of KC.

Monocyte-derived macrophages (MoMF) are a well-studied cellular population in the liver. During liver injury, outside monocytes get recruited to the liver, wherein they take on a macrophage-like phenotype with similar functions. Increasingly, it is being recognized that these monocytes fit one of two very different roles. Classically activated M1 or proinflammatory macrophages generally secrete pro-inflammatory cytokines, are Ly6C^hi^, exacerbate inflammation, and in some cases contribute to cell death [52]. Alternately activated M2 or anti-inflammatory type macrophages secrete large amounts of anti-inflammatory and pro-resolving cytokines like interleukin-10 (IL-10), are stimulated by IL-4, and serve to remove necrotic debris and resolve inflammation in preparation for regeneration [52,53]. Accumulation of M1 type macrophages in the liver would indicate monocytes could potentially cause inflammatory liver injury; whereas accumulation of M2 macrophages would suggest wound healing is beginning to occur and the injury phase is declining [54]. MoMF express the same subset of enzymes necessary for production of oxidative stress as KCs, and are hypothesized to produce toxicity in the same fashion.

Finally, it should be noted that despite its acknowledged roles in multiple disease states, modulation of the innate immune system, especially endogenous KCs, is risky as a therapeutic target. The primary purpose for KCs and other innate immune cells remains the clearance of bacteria. In patients with alcoholic hepatitis, blockade of TNF-α signaling resulted in increased risk of sepsis, and caused excess mortality [55,56]. Similarly, despite the fact that acute liver failure patients suffer from dramatically reduced liver function, a large percentage of patients do not die of hepatic insufficiency, but rather from sepsis associated with liver failure. Given this information, blockade of inflammation remains a controversial target at best in liver disease, and should only be pursued in patients without an active infection, or evidence of systemic inflammatory response syndrome (SIRS). Moreover, more specific therapeutic targets are needed that could potentially reduce the adverse effects of major immune activation, without inhibiting host defense. In spite of this, understanding sterile inflammation in different models is imperative to understanding disease progression and mortality in patients.

#### 2.3.2 Neutrophils

Neutrophil-mediated solid organ injury has been studied extensively in liver and other organs, and requires multiple steps [2,57]. Neutrophil recruitment is necessary, and in high numbers neutrophils can generate sufficient oxidative stress to kill hepatocytes [58]. This is carried out by production of cytokines and chemokines in both mice and humans. Next, neutrophils have to adhere to the vasculature and extravasate into the parenchyma [59,60]. In most organs, neutrophils roll along post-capillary venules through interactions between selectins on endothelial cells and their ligands expressed on neutrophils [61]. Expression of these molecules is regulated by cytokines, and thus cytokines serve not only to recruit neutrophils, but to promote their attachment to the endothelium for transendothelial migration. However, in the liver, only limited neutrophil rolling is present in post-sinusoidal venules [62,63]. In contrast, most neutrophil extravasation occurs from the sinusoids [64]. Both E-selectin [36,65], and their ligands [66], have been suggested to be involved in neutrophil mediated liver injury in different models, indicating E-selectin may still be involved in neutrophil transendothelial migration in sinusoids. Alternatively, the hepatoprotection by blocking selectins may be a secondary effect caused by reduced injury in the intestine in certain models such as ischemia-reperfusion injury [67]. Furthermore, it was hypothesized that P-selectin-dependent platelet accumulation during hepatic ischemia-reperfusion may cause microvascular perfusion failure and facilitate neutrophil accumulation in sinusoids [68,69]. However, the pathophysio-logical role of platelet-neutrophil interactions has recently been questioned [70].

Independent of the controversial role of selectins in the liver, neutrophil transmigration involves mainly interactions of LFA-1 (CD11a/CD18) or Mac-1 (CD11b/CD18) on neutrophils and ICAM-1 on sinusoidal endothelial cells [71]. Once neutrophils transmigrate past the endothelium, interactions between β_2_ integrins (LFA-1/Mac-1) and ICAM-1 on the target cells mediate neutrophil adhesion to target cells [72,73] including hepatocytes [74]. Firm adhesion to hepatocytes is required for toxicity as this triggers the long-lasting oxidant stress and the diffusion of ROS into hepatocytes [2,57]. Furthermore, local anti-proteases nullify the toxic potential of neutrophil-derived proteases without very close physical proximity [75]. Neutrophil cytotoxicity requires upregulation of CD11b on neutrophils, and expression of the Mac-1 complex on the cellular surface of neutrophils [72], in addition to ICAM-1 upregulation on hepatocytes [76,77]. Blockade or deletion of either adhesion component results in a significant decrease in neutrophil toxicity in the liver [32,35,41] as does deletion of ICAM-1 [76-78]. As such, CD11b expression at the cellular surface is a common indicator of neutrophil activation [79]. Finally, when firmly adhered, neutrophils are capable of killing hepatocytes through degranulation and release of a number of cytotoxic species [80,81]. Neutrophils produce ROS through the NADPH oxidase system, which catalyzes the formation of superoxide by transferring electrons from NADPH to molecular oxygen and initiates toxicity [81]. Superoxide dismutates spontaneously or catalyzed by superoxide dismutase (SOD) into molecular oxygen and hydrogen peroxide, which can be toxic to hepatocytes [58]. In addition, neutrophils are the sole producer in mammalian biology of the enzyme myeloperoxidase.

Myeloperoxidase is present in neutrophil granules in the interior of the cell. Upon neutrophil activation, this enzyme is released into the local space where it reacts with chloride anion and hydrogen peroxide produced from NADPH oxidase to generate hypochlorous acid, a potent oxidant [82]. Hypochlorous acid is highly toxic and rapidly adducts tyrosine, which causes significant oxidative stress and protein dysfunction [83]. These adducts are readily identifiable with antibodies against chlorotyrosine, and are present in a number of models of neutrophil mediated liver injury [32,78,81,84]. Additionally, neutrophils express elastase in their azuophilic granules, which can result in cellular dysfunction in some disease, such as emphysema of the lungs [85]. Elastase is a serine-protease thought to be involved in breakdown of extracellular matrix for neutrophil movement. Neutrophil elastase has been suggested to potentially be involved in liver injury [86]; although, mechanisms of how elastase could damage hepatocytes have not been elucidated, nor has there been any update on how elastase escapes the effect of local anti-proteases in the liver [75].

Recently it was identified that neutrophils are capable of secreting a mixture of granule proteins and neutrophil DNA, that form a mesh-like fibrous network known as a neutrophil extracellular trap (NET) [87]. These traps have a clear role in pathogen removal, wherein pathogens are trapped within the fibrous network and are subsequently cleared [87,88]. While this process is unequivocally important in immune function, whether or not NETs can serve as a cause of tissue injury or tissue pathology is poorly characterized. NET formation during sterile inflammation has been noted in multiple models [89,90]. NET formation may have a role in ischemic liver injury, as NETs are formed in ischemic liver lobes and treatment with DNase I both reduced liver injury, and reduced NET formation [91]. This likely occurs through a HMGB1- TLR4 mediated pathway, indicating sterile inflammation can serve as an activator of NET formation independent of bacteria [91]. More research is required in this area, along with more specific ways of targeting NET formation, to fully understand how NETs themselves might drive pathogenesis and damage hepatocytes.

Given these well-established mechanisms of neutrophil cytotoxicity in the liver [57,92], whether or not neutrophils directly cause injury in a model can be assessed by interventions that target these mechanisms. If neutrophils were causing injury, the above steps would happen prior to the major development of injury, biomarkers of neutrophil-mediated liver injury such as chlorotyrosine staining, CD11b upregulation, and localized neutrophil recruitment would be prevalent, and knockout of the critical components such as CD11a, CD11b, CD18, ICAM-1, LFA, or other adhesion molecules would provide protection in relevant models.

#### 2.3.3 Other inflammatory liver cells

In addition to the populations of macrophages and recruited neutrophils, the liver also contains a large population of natural killer (NK) cells of various types including: NK cells, NKT cells, and γδT cells [93]. Natural killer cells produce a number of cytokines that can exacerbate inflammation, but primarily are known for secreting interferon-gamma (IFN-γ) and TNF-α [93]. NK cells have cytotoxic activity as their granules contain a number of cytotoxic proteases that can directly perforate cells causing cell death, and they can also present pro-apoptotic proteins such as Fas ligand and CD40L [94]. NK cells have been implicated as potential players in the inflammatory environment of multiple types of acute liver injury including cholestatic liver diseases [95], APAP-induced liver injury [96], and hepatic ischemia-reperfusion injury [97], although the most common explanation is not as an effector cell, but rather as a stimulant for increased pro-inflammatory cytokine production. The role of NK cells in APAP induced liver injury has been particularly controversial with groups attaining opposite results. Jα18 mice that are deficient in Vα14iNKT cells are also resistant to APAP induced liver injury but this was attributed to changes in metabolism of APAP by the Jα18 mice that preferentially detoxified APAP [98]. These results were also challenged though as studies with CD1d knockout and Jα18 mice later indicated these mice underwent more severe injury due to stabilization and subsequent increases in CYP2E1 levels and thus greater biotransformation of APAP to its reactive metabolite [99]. As such, there is considerable debate in the field as to what the role of NK/NKT/iNKT cells truly is. Additional research is ongoing in this area as to whether or not subpopulations of these cells can potentially modulate the inflammatory environment during acute liver injury.

Dendritic cells have also been implicated in sterile inflammation in the liver. Dendritic cells are antigen presenting cells associated with the adaptive immune system that bridge the gap between innate immunity and adaptive immunity by presenting antigen material to local T cells [100]. The role of dendritic cells in acute sterile inflammation has not been well explored. There is some evidence that depletion of dendritic cells exacerbates APAP-induced liver injury although the mechanism through which this occurs is not confirmed [101].

## 3. Sterile inflammation – models

It is difficult to exactly duplicate a clinical liver disease in mice. Despite this, a number of models have been developed that approximate the clinical liver disease present in patients. Many of these, such as APAP-induced liver injury and hepatic ischemia-reperfusion injury are high fidelity models that have informed clinical practice and dramatically sped up the rate of identifying potential therapeutic targets. These models remain imperative to our understanding of the associated human conditions and, as such, defining mechanisms of injury and inflammation in these models accurately is necessary for clinical advancement. Herein, we will discuss sterile inflammation in the context of multiple diseases in an attempt to understand how inflammation affects liver injury.

## 4. Hepatic ischemia-reperfusion injury

**Figure 1:**
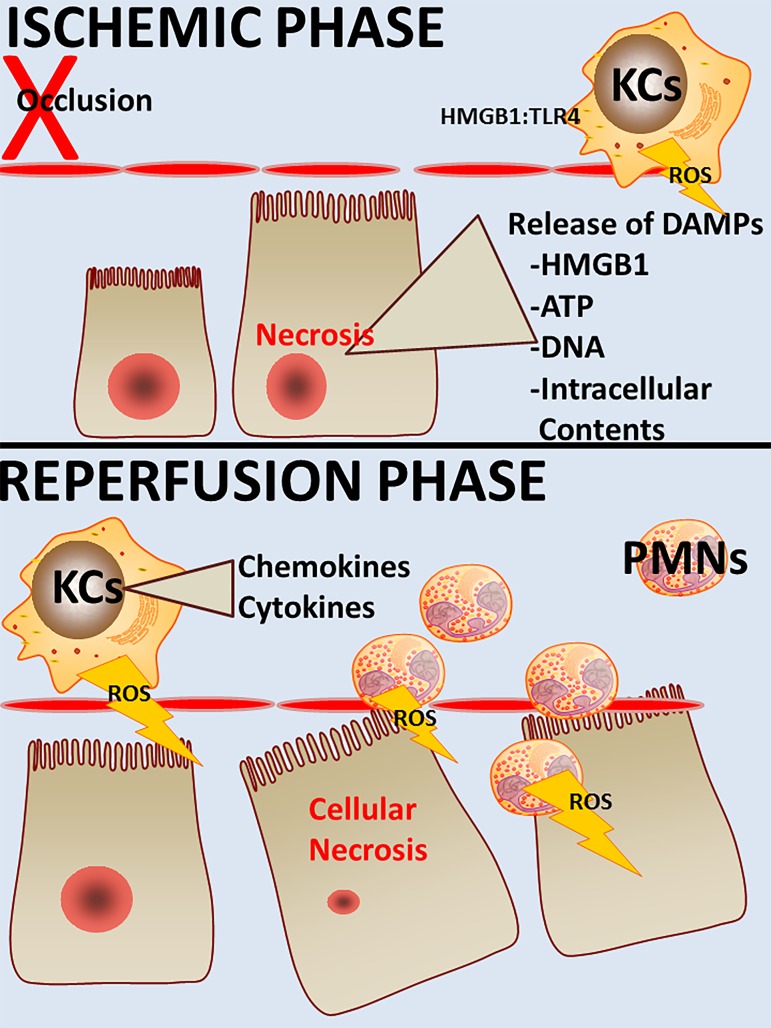
A simple model of ischemia-reperfusion injury. In the ischemia phase (top), occlusion of the blood flow causes hepatocyte swelling and release of intracellular contents that activate local Kupffer cells that express TLR4. This causes release of ROS from KCs. In the reperfusion phase, localized production of chemokines and cytokines by KCs recruit and activate neutrophils. These neutrophils exacerbate the ROS produced by KCs. KC – Kupffer cell, PMN – neutrophil, HMGB1 – high mobility group box-1, ROS – reactive oxygen species, DAMPs – damage associated molecular patterns, ATP – adenosine triphosphate,

Liver transplantation and liver resection are becoming increasingly common interventions for patients with advanced liver disease or liver tumors, respectively. Both transplantation and resection require clamping the hepatic portal triad, and thus induce ischemia, followed by unclamping and reperfusion of the liver. This procedure can cause cell injury during ischemia (due to lack of oxygen and absence of blood flow) and during reperfusion (due to the reintroduction of oxygen and blood flow). The overall pathophysiology of hepatic ischemia-reperfusion injury is complex [39,102-104]. Hepatic ischemia-reperfusion injury is a classical and widely accepted example of sterile inflammation and inflammatory liver injury [57,102,103] (Figure 1). The basic mechanisms that control liver injury in this model serve as a guide.

### 4.1 Ischemic liver injury

Clamping of the vasculature leads to immediate changes in hepatocytes. Primarily, the lack of oxygen shifts metabolism away from oxidative phosphorylation and towards glycolysis. This results in depletion of ATP, and a dramatic increase in intracellular constituents such as lactate, increased intracellular osmolarity, and reduced pH due to accumulation of H^+^ cations [100,105]. Eventually, these cells will undergo necrosis due to accumulation of calcium, and breakdown of membrane potential, however, there is no formation of reactive oxygen species [107,108]. Under realistic clinical conditions during elective surgery, the ischemic time will be limited in order to avoid severe ischemic injury. Nevertheless, hepatic ischemia together with early events during reperfusion, which include cell swelling caused by hyperosmolarity in the cytosol due to the accumulated metabolites, will lead to cellular stress and release of intracellular content [109]. Once the accumulated metabolites are either metabolized or flushed out, the swelling will subside. If there was no mitochondrial damage during ischemia, there will be limited intracellular oxidant stress during reperfusion [108]. On the other hand, if there was ischemic damage, the intracellular post-ischemic oxidant stress will be derived from xanthine oxidase, and more importantly, from damaged mitochondria [110]. In patients, prolonged hepatic ischemia can trigger ischemic hepatitis, which has a very high mortality rate [111]. Biomarkers indicate that ischemic hepatitis involves severe necrosis with extensive mitochondrial damage [112].

### 4.2 Reperfusion injury – initiation

A majority of cell death occurs after reperfusion [113]. Hepatocytes are generally resistant to ischemia for long periods, in part due to the fact that the mitochondrial membrane permeability transition pore (MPTP) opening can be reversible [114]. However, once re-oxygenation begins, hepatocyte leakage and mild necrosis releases a number of DAMPs such as HMGB1 [115], and mitochondrial DNA [14]. HMGB1 release occurs as early as one hour post-reperfusion, and continues to increase for up to twelve hours later [116]. HMGB1 activates TLR4 on KCs [115] and hepatocytes [117]. In addition, complement is activated during this period which directly activates KCs to generate reactive oxygen and complement components are potent neutrophils activators [41]. Thus, inhibition of complement activation or depletion of complement factors is highly protective against the injury [41]. KCs then produce ROS that can damage hepatocytes [41,118]. In addition, cytokines such as TNF-α and chemokines promote neutrophil recruitment [119,120]. In murine models, this also includes activation of the Nalp3 inflammasome through an apoptosis-associated speck-like protein (ASC), which exacerbates further inflammation [121]. Inflammasome activation may occur through TLR9 in this model, although a better characterization of inflammasome activating factors such as histones are needed [122]. A number of experimental interventions support the idea that KCs are critical for the initial injury and the progression of the inflammatory response [102]. The predominant oxidant stress occurs largely in the vasculature and not in parenchymal cells during reperfusion, indicating that hepatocytes do not contribute directly to the oxidative stress [40]. Inactivation of KCs dramatically reduces injury and vascular oxidant stress [40]. Preconditioning of the liver by pulsing short bursts of ischemia and reperfusion induces a number of anti-oxidant genes that are protective [123]. Administration of glutathione is also protective through the scavenging of the vascular oxidant stress [124-126]. A number of different pharmacological agents have been shown to act through either reduction of KC- mediated oxidative stress or through a direct action on KC activation [39,127]. After the initial oxidative stress from KCs, secreted cytokines, chemokines and activated complement factors further recruit neutrophils which produce more cytokines formation and more oxidant stress, leading to a vicious cycle of continued inflammation and injury [41,118-120,128,129].

### 4.3 Reperfusion injury – a second stage

Neutrophils exacerbate ischemia-reperfusion injury in the liver [35]. Neutrophil accumulation in the post-ischemic liver lobes is obvious and dramatic as early as three hours post-reperfusion [41]. Of note, neutrophils begin to upregulate CD11b as early as one hour post-reperfusion, consistent with a neutrophil mediated liver injury [130]. Inactivation of neutrophils with a Mac-1 monoclonal antibody is protective against the injury, even when administered only 30 minutes before the onset of ischemia [130]. Treatment of rats with an antibody against ICAM-1 is also protective [77], even in steatotic livers that are more prone to cell death after transplantation [126]. Protection against hepatic ischemia reperfusion with a number of agents is correlated strongly with a reduction in neutrophil recruitment or function [39]. Generally, inhibition of upstream mediators of inflammation such as HGMB1 [115], or mitochondrial DAMPs [14, 131], prevents downstream neutrophil accumulation and is highly protective against injury. As such, neutrophils are the likely critical mediator of the second phase of injury. In addition, it has been noted that other inflammatory cells might be involved in the second phase. CD4^+^ T cells also accumulate rapidly in the liver [132]. The primary mechanism through which these cells injure hepatocytes and exacerbate injury is likely through enhanced recruitment of neutrophils in an IL-17 dependent fashion [133]. Interferon regulatory factor 3 (IRF3) might be the critical link between recruitment of T-cells and the initial oxidant stress, as IRF3^-/-^ mice are strongly protected against hepatic ischemia reperfusion injury in an IL-17 dependent mechanism [134].

Cytokine expression profile during this period is integral to the injury as activation of CXCR4 [135], and CXCR2 [29] are both detrimental to liver health; whereas, a number of other interleukins and cytokines such as IL-33 [136], and IL-37 [137], as well as other KC-derived mediators such as secreted leukocyte protease inhibitor (SLPI) reduce and control excessive inflammation [138]. The inflammation is eventually resolved through these anti-inflammatory processes, largely via production of IL-10 and activation of NF-κB [139] as well as production of IL-4 and activation of signal activator and transducer 6 (STAT6) [140] and production of IL-6 [141]. Resolution of hepatic ischemia-reperfusion injury is critical to recovery from both resection and liver transplantation, which is apparent in clinical practice by the substantial number of anti-inflammatory and immuno-modulatory drugs given in both settings [142].

## 5. Acetaminophen-induced liver injury

By far the most controversial topic in the field of sterile inflammation is the role of inflammation in APAP-induced liver injury. Despite the fact that there has been an effective antidote for over 30 years to prevent APAP-induced liver injury in early presenting patients, there still is no antidote for preventing late-stage APAP hepatotoxicity, or APAP-induced acute liver failure (ALF). Administration of N-acetylcysteine (NAC) replenishes glutathione (GSH) levels, which can first scavenge the reactive metabolite N-acetyl p-benzoquinone imine (NAPQI) and later support ROS detoxification [143]. NAC is most effective at early time points. After APAP metabolism is complete, the efficacy of NAC is diminished [144]. To address this concern, many have proposed the idea that there is a second phase of injury associated with APAP overdose that is dependent on inflammatory cells, predominantly focused on KCs, neutrophils and monocytes [30,86,96,145-147]. Despite the obvious appeal and some experimental support, there is a considerable amount of evidence that also argues for the contrary [5,148].

### 5.1 Acetaminophen induced liver injury – intracellular oxidative stress and the initial phase of toxicity

The initial phase of toxicity has been extensively studied over the last 40+ years. Foundational work was performed by Mitchell and colleagues when they demonstrated that APAP overdose rapidly depletes GSH, causes APAP protein adducts and leads to hepatocellular necrosis [149]. Subsequent work has demonstrated a number of the intracellular signaling mechanisms responsible for both metabolism and the toxicity.

APAP is rapidly glucuronidated by UDP-glucuronosyltransferase 1A1 and sulfated through sulfotransferase 2A1 [150]. During an overdose, only the sulfation but not glucuronidation pathway is saturated [151]. While neither of these processes produces a toxic metabolite, oxidation by cytochrome P4502E1 results in the formation of NAPQI [152], which can adduct proteins and trigger a profound mitochondrial oxidative stress [153]. The exact nature of which proteins become adducted remains a question in the field, but what is understood is that this intracellular oxidative stress mediates a substantial portion of the subsequent injury through a number of mechanisms. Foremost, the oxidative stress triggers activation of the c-Jun N-terminal kinase (JNK) pathway, and results in phosphorylation of JNK, and translocation of JNK to the mitochondria [154]. A number of other pathway members are also activated including Sab, MLK3, and Ask1, and also contribute to the injury process [155-157]. Knockout or pharmacological inhibition of these proteins is protective, as is knockout or pharmacological inhibition of JNK itself [158]. In addition to JNK activation, the oxidative stress activates the nuclear factor (erythroid-derived 2)-like 2 (Nrf2) signaling pathway [159]. This results in upregulation of a number of cytoprotective molecules that mediate autoprotection upon further APAP dosing [159]. Knockout of Nrf2 dramatically exacerbates injury [159], and knockout of the Nrf2 binding factor Kelch like-ECH-associated protein 1 (Keap1) is highly protective against APAP-induced liver injury [160]. This same oxidative stress also activates a number of different B-cell lymphoma-2 (Bcl-2) family proteins including Bcl-2 associated X-protein (bax) and BH3 interacting death domain agonist (bid) [161,162]. Both of these factors translocate to the mitochondria where they form pores in the outer mitochondrial membrane and help to initiate the mitochondrial permeability transition (MPT). Simultaneous to these events, is activation of the receptor interacting kinase 1/receptor interacting kinase 3 (RIP1/RIP3) pathway [163-165]. In the classical necroptotic pathway, these proteins form a complex called the ripoptosome that can activate mixed lineage kinase domain-like (MLKL), which then translocates to the cell membrane and forms membrane-disrupting pores leading to necrotic cell death [166]. However, recent studies showed that MLKL^-^/- mice are not protected against APAP hepatotoxicity suggesting that APAP-induced cell death is not a necroptotic mechanism [165,167]. Although there seems to be no role for MLKL and thus direct necroptosis in APAP-induced liver injury, yet knockout of either RIP1 or RIP3 seems to be protective [163,165]. While the current role of these proteins is not well understood in APAP toxicity, it is believed that this complex might have other functions that promote cytotoxicity. Regard-less, the primary event that occurs subsequent to all these events is MPT formation in the mitochondria [168]. JNK translocation to the mitochondria dramatically enhances the mitochondrial oxidative stress and stimulates mitochondrial dysfunction and pore opening [169,170]. This causes complex cellular dysfunction and reduces ATP stores for the cell [171]. Moreover, the mitochondrial oxidative stress results in leakage of mitochondrial membrane proteins such as apoptosis-inducing factor (AIF), and endonuclease G [172,173]. Both proteins are nucleases and translocate the nucleus where they directly attack DNA and eventually cause nuclear DNA lysis and cell death [173]. The primary oxidative stress and GSH depletion occurs within the first two hours, and the secondary oxidative stress occurs for the following 4-12 hours. During this secondary period, stimulation of autophagy also occurs, and autophagy can protect against APAP induced liver injury by removing damaged mitochondria that leak cytotoxic proteins [174,175]. Autophagy also removes protein adducts which reduces the initial harmful stimuli and further protects against future cell death [176]. This is especially apparent in cells distal from the central vein area where the primary site of toxicity is, as these cells express lower quantities of CYP2E1, generate fewer adducts, and undergo less oxidative cell death [177]. This initial phase of toxicity is supported by dozens of papers, interventions, and mouse models that all point towards the idea that protein adducts serve as a central source of oxidative stress that targets the mitochondria and eventually causes cellular necrosis due to DNA lysis and failed mitochondria.

### 5.2 Sterile inflammation – a second phase of injury after APAP overdose?

#### 5.2.1 Neutrophils

The idea of a second phase of injury has been controversial in the literature. The primary hypothesis that has been put forth many times is that recruited inflammatory cells, predominantly monocytes and neutrophils, exacerbate the initial injury through the release of reactive oxygen species and other cytotoxic compounds consistent with sterile inflammatory liver injury. This occurs after the initial oxidative stress, typically reported between 6 and 12 h after the initial overdose of APAP. Studies in favor of this hypothesis have presented a number of points that indicate depletion of inflammatory cells, depletion of specific inflammatory mediators and chemotactic factors, or pharmacological inhibition of specific mediators of the injury is protective against acetaminophen-induced liver injury [30,86,96,142,178-182]. Moreover, during the inflammatory injury phase there is accumulation of a number of these factors including the release of DAMPs, chemokine and cytokine formation, substantial neutrophil and monocyte recruitment, and co-localization of these factors with areas of necrosis [30,86,96,142,178-182]. As such, at first glance, the combination of these studies strongly supports this idea and argues in favor of a sequential sterile inflammatory response that exacerbates APAP-induced liver injury.

Despite the considerable evidence presented in these studies, there are a number of unresolved issues with this hypothesis. Foremost, there is still a substantial controversy over the obtained results in this field, with many studies not being repeatable by others laboratories [5]. The first report of a potential neutrophil-mediated cytotoxicity after APAP overdose roundly rejected the hypothesis as there was no evidence in favor of upregulation of CD11b, shedding of L-selectin, or other evidence of neutrophil activation during the toxicity phase, nor was there protection when a CD18 antibody was administered to reduce recruitment, transmigration and cytotoxicity of neutrophils [34]. This was challenged by a subsequent study that found that depletion of NK/NKT cells reduced liver injury [96], with the same authors later proposing that the neutrophil was the main mediator of the second phase of APAP-induced liver injury [96]. Both of these studies have contrary reports indicating that the results depend on a questionable experimental design. The protective effect of NK/NKT cell depletion appeared to depend on the presence of dimethyl sulfoxide (DMSO), i.e. in the absence of DMSO as solvent for APAP no effect of NK/NKT cells was detectable [183]. In addition, neutrophil depletion is only effective against APAP toxicity when the neutropenia-inducing antibody is given as a 24 hour pre-treatment [142,145,184], but not when the antibody is administered shortly after APAP [184]. Importantly, it takes only 30 minutes to an hour to completely deplete neutrophils using the Gr-1 antibody that was used in these studies [185]. Interestingly, pre-treatment with the neutrophil-depleting antibody causes a pre-conditioning effect which activates a number of cyto-protective genes [186]. Use of these neutrophil-depleting antibodies has been repeated in subsequent studies, with the authors coming to the same conclusions despite the obvious problems present in this system [182]. If the Gr-1 antibody was truly protective due to its neutropenia effect, then administration just before the initial toxicity would also be effective; however, the Gr-1 antibody is clearly not protective when administered shortly after APAP administration but before any evidence of liver injury [184]. It has been noted though, that neutrophil activation as measured by CD11b expression and priming for ROS occurs largely after the development of liver injury, both in mice and more importantly, in man [184,187,188]. Moreover, inhibition of NADPH oxidase activity by either chemical inhibitors or deficiency of the gp91phox subunit, which completely inhibits hypochlorous acid formation as it stops the production of superoxide, is not protective [184,188]. In addition to anti-CD18 antibody administration not being effective [34], gene deficiency of CD18 is also ineffective, once again showing that there is no neutrophil-mediated cytotoxicity [187]. Similarly, it was proposed that the deletion of the CXC receptor 2, which binds neutrophil chemoattractant chemokines, was protected [189], although this was supposedly not through inhibition of neutrophils but expression of anti-apoptotic genes. However, apoptosis as mode of cell death during APAP hepatotoxicity has been roundly rejected on multiple occasions [190,191]. It has also been proposed that neutrophils mediate toxicity after APAP overdose through release of elastase [86], a serine protease whose primary purpose is to enhance transendothelial migration through the breakdown of extracellular matrix components such as elastin. Elastase is largely present in azurophilic granules present in neutrophils. The authors of this manuscript failed to assess neutrophil activation, a required component of degranulation, and only reported that mice with elastase-deficient bone marrow cells were protected with no explanation as to how the actual cytotoxicity might occur [86]. Furthermore, in our hands, elastase-deficient mice were not protected against APAP toxicity, nor was a pharmacological inhibitor of elastase protective (Jaeschke and Woolbright, unpublished). In addition to these direct interventions against neutrophils, other studies that affected the availability of various DAMPs such as uric acid, or complement, had a substantial effect on hepatic neutrophil accumulation but did not attenuate liver injury [192,193]. Given the totality of this data, it is clear that there has yet to be a study to conclusively demonstrate the role of neutrophils as a feed-forward amplification loop of APAP- induced liver injury. The current data surrounding the role of neutrophils is largely based on experimental approaches that have been questioned. At the present time, there is no conclusive evidence for a relevant role of neutrophil cytotoxicity in this model and most importantly in patients [188].

#### 5.2.2 Macrophages and monocytes

Similarly, there are multiple reports on the efficacy of reducing either endogenous macrophage function or recruited monocyte function; however, these reports came to drastically different conclusions despite the fact the same strains of mice and same chemicals were used to derive these results. It was reported that pre-treatment with gadolinium chloride, a compound that inactivates KCs and attenuates ROS formation [96], prevented almost 100% of APAP toxicity [178]. However, these findings have never been confirmed by others [194-196]. Gadolinium chloride treatment may have additional off-target effects and its use has caused controversy in other models [43,197]. Moreover, using a separate approach to eliminate KC activity, clodronate liposomes, caused the opposite result, i.e., aggravated APAP hepatotoxicity [195]. In addition, mice deficient in the gp91phox gene, a component of the NADPH oxidase, showed the same oxidant stress and liver injury as wild type animals [188,198]. Taken together, these findings suggest that KCs are not directly involved in the injury.

Of note, monocytes and MoMF have also been investigated repeatedly with highly variable results [27,28,145,199-201]. Despite using the same mice, these groups obtained opposite results in regard to the role of the macrophage recruitment receptor CCR2 in APAP-induced liver injury with groups finding no differences in injury between CCR2^-^/- and wild type mice [27,28], groups finding exacerbation of injury in CCR2^-^/- mice compared to wild type animals [145,199] and one group finding protection against the injury in CCR2^-^/- mice [30]. Variable results such as this remain unexplained in the literature. Importantly, results obtained with MCP-1^-^/- mice matched the findings in the CCR2^-^/- mice in one of the studies, indicating that CCR2/MCP-1 signaling is likely not involved in the injury [27]. In support of the animal studies, the role of macrophages has been investigated in patients [202-204]. Macrophages display a distinct M2 phenotype in APAP overdose patients [204]. This applies to both endogenous macrophages, which are proliferative after injury, and bone marrow-derived monocytes that are recruited via CCL2/MCP-1 [204]. These data correlate with data from the mouse, wherein it was shown that recruited macrophages are also of the M2 phenotype and more importantly, tissue regeneration and repair after APAP overdose was delayed in CCR2^-^/- mice [27,28,200]. Moreover, high levels of secretory leukocyte protease inhibitor released from hepatocytes and macrophages inhibit immune function and increase susceptibility to sepsis and infection in APAP overdose patients [204]. Given this anti-inflammatory, pro-regenerative phenotype, macrophages are likely a key mediator of recovery, and preventing their recruitment and inactivation would be ill-advised. Even still, more reproducible effects in preclinical models are needed before any reliable conclusions can be drawn, and pitfalls in the experimental systems need to be identified and avoided.

#### 5.2.3 The inflammasome

Although the evidence in favor of any specific inflammatory cell being the mediator of APAP-induced liver injury is limited in comparison to the evidence against it, there is also considerable evidence both for and against the role of specific mediators of inflammation, including a number of chemokines and cytokines. One of the foremost ideas in the field is that the inflammasome is primed through DAMPs binding to toll like receptors, e.g. TLR4 and TLR9, and actually activated by other DAMPs such as ATP, and that this activation mediates the injury through production of IL-1ß [3,28,29,181,182]. In support of this, there are obvious increases in serum mitochondrial DNA [13], and serum ATP [205], after APAP overdose, and there is a modest increase in pro-IL-1ß expression and serum IL-1ß levels after APAP overdose -179, 180, 206]. In addition, the formation of IL1β but not pro-IL-1β (IL-1β mRNA) is inhibited by a caspase inhibitor [206]. Furthermore, the supposed inhibition of TLR4 by benzyl alcohol can suppress IL-1ß signaling and protect against the injury [180]. Finally, there is reported protection in a number of models including NLRP3^-^/-, ASC^-^/-, P2XR7^-^/-, and TLR9^-^/- mice in addition to protection by pharmacological inhibition of TLR9, or treatment with apyrase, or treatment with DNase I, to breakdown extracellular DNA and prevent TLR9 activation [21,179-182,205]. However, some of these reports also are controversial as attempts to repeat much of these data have failed. Foremost, protection against injury with NLRP3^-^/-, ASC^-^/-, caspase-1^-^/-, IL-1R-/- mice and treatment with caspase inhibitors failed to provide protection in subsequent studies [206,207]. Administration of IL-1ß directly in high doses did not yield any increase in toxicity, despite an increase in neutrophil recruitment [206,207]. Treatment with P2XR7 pharmacological antagonist yielded more protection than the corresponding gene knockout of P2XR7 [21], and the inhibitor was subsequently shown to inhibit APAP metabolism, and thus its protective effects were independent of its alleged pharmacological effects on inflammasome activation [208]. Furthermore, evidence that extracellular ATP directly exacerbates APAP- induced cell death [205] has also been un-repeatable in both rodent and human hepatocytes [209]. Most importantly, human plasma samples from APAP overdose patients did not have any evidence of enhanced IL-1ß formation at any point over the first seven days of injury [5]. Thus, the inflammasome is not a likely a therapeutic target for preventing APAP toxicity, and its role in human patients is limited at best.

#### 5.2.4 Interleukins

In addition to IL-1β, APAP exposure causes upregulation of several additional pro- and anti-inflammatory members of the interleukin family. Knockout of the IL-10 gene exacerbated APAP-induced liver injury [210]; however, this increased injury was linked to induction of inducible nitric oxide synthase in the absence of IL-10 and not an immune cell-mediated injury, indicating the increased injury was due to an effect on intracellular signaling mechanisms as APAP overdose causes extensive peroxynitrite formation in the mitochondria [211,212]. Knockout of IL-6 did not affect the injury, but did increase the rate of hepatocyte regeneration [213]. This may be due to signaling interactions with macrophages as inhibition of monocyte recruitment slows the rate of regeneration [28]. The role of IL-4 has become controversial as groups have attained opposite results in different strains of mice [214,215]. IL-4 is protective in C57BL/6J mice as it sustains increases in glutathione synthesis and recovery that are responsible for detoxification of oxidant stress in APAP toxicity [214], and thus IL-4^-^/- mice develop more severe injury. IL-4 administration in this model reversed the deleterious effects [214]. Others have recently reported that in the Balb/C mouse IL-4 exacerbates injury by enhancing inflammation and glutathione depletion [215]. This was in direct contrast to the previous results, although no attempts were made to explain these opposite findings. Since the article by Ryan and coworkers used multiple approaches to assess the role of IL-4, it is likely that this cytokines is beneficial by promoting hepatic GSH recovery [214]. Nevertheless, it needs to be kept in mind that the effect of IL-4 may be strain-dependent.

## 6. Liver injury during obstructive cholestasis

Cholestasis is caused by a blockage in bile flow from the liver to the intestine. It occurs either within the internal bile ducts of the liver, or in the external bile duct that connects the liver to the intestine. Regardless of the location, substantial liver injury occurs when animals or human patients undergo severe obstructive cholestasis. Inflammation is a noted aspect of murine models such as bile duct ligation (BDL) in rats [216], or mice [32], the multi-drug resistance protein 2 (MDR2) knockout mouse [217], and in human cholestasis patients of various etiologies [51]. Previous studies have indicated that cholestatic liver injury is mediated by both bile acid accumulation [218], and through inflammation, with both KCs [43], and neutrophils [32] being critically involved. One of the remaining questions in the field is which of these processes stimulates the initial cell death, and which mechanisms and cell types are then responsible for cell death. Herein, we will discuss these mechanisms with a focus on the BDL model, due to its prevalence in the literature, while also focusing on human patients.

### 6.1 Cholestatic liver injury – bile acid induced toxicity

When hepatocytes are exposed to sufficient concentrations of specific bile acids, they can undergo a variety of changes including apoptosis [218], necrosis [51,219], dramatic upregulation of cytokines such as MIP-2 and mKC [25], and activation of signaling pathways through G-protein coupled bile acid receptor (TGR) and farnesoid X receptor (FXR) [220,221]. With the exception of the activation of the above signaling pathways, which seem to occur in both human and rodent cells, many of these other changes are largely dependent on the origin of hepatocytes and the associated, individual bile acid species. Hepatocytes derived from the rat rapidly undergo apoptosis upon exposure to high concentrations of glycochenodeoxycholic acid (GCDC), a bile acid that is predominantly found in humans [51,222]. While apoptosis is not considered as pro-inflammatory as necrotic cell death, due to the lack of release of DAMPs, it has been noted that apoptosis can cause inflammation through serving as a danger signal itself, and through activation of local phagocytic macrophages [43,52]. In contrast, a number of other mammalian cells, including humans, are highly resistant to GCDC-induced apoptosis and require high concentrations of bile acids to initiate cell death [51,219,223]. Instead, hepatocytes from both humans and mice undergo frank necrosis after exposure to high concentrations of bile acids and release DAMPs such as HMGB1 [51]. Moreover, the relative toxicity of individual bile acids is highly variable, and dependent on their side chains, and to which amino acid they are conjugated [224]. The overwhelming majority of bile acids in cholestatic mammals are conjugated to either taurine or glycine [225]; however, the degree to which mammals use each amino acid varies greatly [51,226]. This has a number of biological effects, as conjugation to taurine reduces toxicity, but seems to promote inflammation, at least in the mouse [25]. Many of the less toxic taurine-conjugated bile acids, such as taurocholic acid, accumulate significantly in both mice and humans [226], and strongly upregulate and promote a number of pro-inflammatory cytokines in the mouse [25]. Of note though, this interaction is less prominent in either primary human hepatocytes [51], or human HepaRG cells [227]. As such, accumulation of bile acids can affect sterile inflammation in a number of ways: 1) bile acidinduced apoptosis can stimulate inflammation by activating KCs through phagocytosis of apoptotic bodies [43]; 2) bile acid-induced necrosis can stimulate inflammation through DAMP release [51]; 3) bile acids can directly stimulate inflammation without causing cell death through an early growth response factor-1 (Egr-1)-dependent signaling pathway [25]; 4) accumulation of bile acids and biliary pressure can activate signaling molecules such as osteopontin that result in neutrophil recruitment [228]. All of these pathways and mechanisms are contextual, based both on which bile acids are administered or which bile acids accumulate in the model or human patient, and also what sort of rodent model or human patient is being examined.

### 6.2 Bile duct ligation – a consensus model of inflammatory liver injury

BDL results in an immediate increase in biliary pressure that initiates overload of the biliary tracts and causes rupture of the bile ducts and release of bile onto the hepatic parenchyma within 6 hours [229,230]. Neutrophil counts in the liver rise about this same time, and are largely localized to the area immediately surrounding the area of biliary infarction [33]. Concomitantly, neutrophil activity rises acutely after BDL, and neutrophils from BDL animals have elevated NADPH oxidase activity [216,231]. Previous work from our laboratory has demonstrated that deficiency of the adhesion molecules CD18 or ICAM-1 is 60-80% protective against BDL injury [32,78]. Presumably, this is due to the lack of observable neutrophil extravasation present in the knockout animals [32,78]. Mice deficient in the Fas receptor are also protected against BDL via a substantial reduction in immune activity [232]. Supporting these data, P-selectin glycoprotein-1^-^/- mice with deficient neutrophil attachment are also protected against BDL [66]. Knockout of the pro-inflammatory factor osteopontin is highly protective at early time points suggesting that cleaved osteopontin may be the initial critical chemotactic factor for neutrophil accumulation after BDL [228]. Osteopontin is cleaved by matrix metalloproteinases, and inhibition of these enzymes is also protective [228,233,234]. Expansion of this inflammatory response over time appears to involve Th17 cells as administration of IL-17 worsens inflammation and injury [235], and knockout of cluster of differentiation 279 results in a dramatic decrease in IL-17 producing cells which correlated with protection from inflammation and injury [236]. Multiple models of mice with an attenuated inflammatory response are protected including the *lpr* mouse [232,237], and the Na^+^/H^+^ exchanger regulatory factor^-/-^ mouse [238]. All of these studies point towards a single hypothesis – the inflammation associated with bile duct ligation mediates a significant portion of the injury [19,239].

Despite this, the driving agent for inflammation in the model is still poorly characterized [239] Inflammation after BDL largely occurs at, and around, the point of biliary infarction, that is, the point of rupture of the biliary ducts due to the increased mechanical pressure [33,240]. Given that these points of rupture are also the site of the overwhelming degree of the associated necrosis, it has been hypothesized that bile acids released from the bile ducts directly damage hepatocytes, resulting in DAMP release and inflammation [237]. Initially, the hypothesis was that bile acid accumulation in the BDL model resulted in apoptosis, which drove the inflammatory response and subsequent neutrophil infiltration which exacerbated the injury and the fibrosis [237]. This was subsequently questioned as there is a lack of apoptosis in the BDL model, and the injury proceeds predominantly through necrosis [33,240]. Apoptosis of normal hepatocytes has only been convincingly shown in rat hepatocytes exposed to high concentrations of GCDC [51,222], however, GCDC is not typically found in rodent models [226]. Attempts at defining a bile acid-dependent cell death in the mouse showed conclusively that concentrations of bile acids that did accumulate in the mouse were largely non-toxic, as taurine conjugated bile acids did not elicit toxicity at concentrations of up to 10 mM, far higher than what was present in serum or bile of cholestatic mice [25,226]. Instead, exposure of primary murine hepatocytes to taurine-conjugated bile acids such as taurocholic acid (TCA) elicited a dramatic increase in CXC chemokine expression and upregulation of ICAM-1 [25], similar to what was observed in vivo after BDL [232,241]. In addition, deficiency of osteopontin resulted in nearly complete protection against necrosis at early time points during BDL, but the protection was not sustained at distal time points [226]. This suggests that cleaved osteopontin is an early chemotactic factor for neutrophils that is released into the parenchyma after BDL. Subsequently, the chemotaxis may be maintained by CXC chemokines generated by bile acid-exposed hepatocytes [239]. Exposure to hydrophobic bile acid does cause significant cellular stress as indicated by mitochondrial dysfunction [242, 243]. This may result in the release of additional local factors responsible for pro-inflammatory mediator generation, as there is substantial HMGB1 release as early as 6 hours post BDL [33]. More studies are needed to better define the initiating factors why neutrophils target damaged or dying hepatocytes during cholestasis, especially in humans.

### 6.3 Obstructive cholestasis in human patients

Cholestasis in humans occurs in various pathophysiologies. Acute obstructive cholestasis induced by gallstones is rarely a chronic concern as patients will commonly present to the hospital rapidly with abdominal pain. The obstruction can be removed by endoscopic retrograde cholangiopancreatography. However, patient populations exist that cannot safely undergo endoscopy, and a greater understanding of cholestasis in patients is required to generate therapeutic options. Acute obstructive cholestasis presents with dramatic increases in alkaline phosphatase, and bilirubin, in addition to increases in serum transaminases. Furthermore, very early studies understood that serum bile acid levels were also dramatically elevated in cholestatic patients [225]. The idea that elevated serum bile acids might induce toxicity was proposed and tested, whereupon it was noted that bile acids induce toxicity in human hepatocytes at concentrations between 500μM and 1mM GCDC, far higher than what is necessary in rats or mice [51]. Human hepatocytes are also highly resistant to taurine-conjugated bile acids, and more importantly, they do not upregulate ICAM-1 or chemokines upon exposure to TCA [51]. A number of subsequent studies have conclusively shown the concentration necessary for toxicity is multiple orders of magnitude above human serum values achieved during cholestasis [51,244,245]; however, biliary concentrations for GCDC and other glycine-conjugated bile acids are commonly in the mM range [51, 246]. As such, it is possible that biliary concentrations of bile acids can directly elicit toxicity. As the characteristic “foamy necrosis” and biliary infarcts present in BDL are also present in human patients, biliary bile acid concentrations may be responsible for the initial toxicity in human patients and subsequent release of pro-inflammatory factors such as HMGB1 [51]. While a sterile inflammatory response during obstructive cholestasis is clearly present in laboratory animal and in human patients, there are obvious differences between the current rodent models and human patients. Further studies are also needed in human patients to more fully define if neutrophils, or other inflammatory mediators, exacerbate the disease.

## 7. Conclusions

A sterile inflammatory response is an important aspect of acute liver injury. As the liver is an innate immune organ by nature, there is a delicate balance between activation, over-activation and hyper-stimulation that is constantly ongoing. Future studies are needed to better define the mechanisms that drive a sterile inflammatory response in various models and the pathophysiological relevance for the progression of the injury and the critical involvement in the process of regeneration and recovery. In addition, the vital importance of inflammation as defense against infection needs to be taken into account. Only a more detailed understanding of these mechanisms in relevant animal models and patients will allow the identification of potential therapeutic targets that can be used to translate these findings in animals to patients without impacting normal host defense functions.
